# Cumulative Risk Assessment: An Overview of Methodological Approaches for Evaluating Combined Health Effects from Exposure to Multiple Environmental Stressors

**DOI:** 10.3390/ijerph9020370

**Published:** 2012-01-26

**Authors:** Ken Sexton

**Affiliations:** Department of Epidemiology, Human Genetics, and Environmental Sciences, University of Texas, School of Public Health, Brownsville Regional Campus, 80 Fort Brown, Brownsville, TX 78520, USA; Email: ken.sexton@uth.tmc.edu; Tel.: +1-956-882-5164; Fax: +1-956-943-5152

**Keywords:** chemical mixtures, combined health effects, cumulative risk assessment, environmental justice, nonchemical stressors, risk analysis

## Abstract

Systematic evaluation of cumulative health risks from the combined effects of multiple environmental stressors is becoming a vital component of risk-based decisions aimed at protecting human populations and communities. This article briefly examines the historical development of cumulative risk assessment as an analytical tool, and discusses current approaches for evaluating cumulative health effects from exposure to both chemical mixtures and combinations of chemical and nonchemical stressors. A comparison of stressor-based and effects-based assessment methods is presented, and the potential value of focusing on viable risk management options to limit the scope of cumulative evaluations is discussed. The ultimate goal of cumulative risk assessment is to provide answers to decision-relevant questions based on organized scientific analysis; even if the answers, at least for the time being, are inexact and uncertain.

## 1. Introduction

When formal methods to estimate human health risk from exposure to environmental agents were first employed by the U.S. Environmental Protection Agency (EPA) and the U.S. Food and Drug Administration (FDA) in the 1970s, the emphasis was on determining the carcinogenic potency of individual manufactured chemicals. Early risk assessments consequently focused on evaluating adverse health outcomes from anthropogenic carcinogens in air, water, and food. The risk assessment processes and procedures that subsequently evolved were strongly influenced by the regulatory attitude prevailing in that epoch, which stressed implementation of national command-and-control strategies and technology-based legal statutes to control pollution on a chemical-by-chemical basis [1,2,3,4]. Over the past 35 years, the vast majority of risk assessments conducted by EPA have concentrated narrowly on individual chemical agents, distinct sources or source categories, and single exposure pathways, environmental media, routes of exposure, and health endpoints [2,3,4]. It is becoming apparent, however, that a more holistic approach is necessary if risk assessment is to remain a relevant and reliable decision-making tool [2,3,4].

The potential for interactions among mixture constituents to produce synergistic effects is well known (e.g., increased risk of lung cancer from combined exposure to tobacco smoke and radon), and EPA has conducted risk assessments for a few chemical mixtures with sufficient epidemiologic evidence (e.g., coke oven emissions, environmental tobacco smoke, diesel exhaust) [5,6,7]. The EPA has also established screening methods to provide rough, first-cut approximations of cumulative health risk from exposure to non-carcinogenic chemicals with the same mode of toxic action (e.g., Hazard Index approach, Margin of Exposure procedure, Toxicity Equivalency Factor method) and to carcinogenic chemicals (e.g., sum individual risks to get total cancer risk from all carcinogens regardless of target site) [5,8,9,10]. Nevertheless, there is a growing mismatch between the broader, real-world questions being asked by decision makers and important stakeholders, and the narrow, limited answers provided by conventional risk assessments [2,4,11,12,13,14,15,16]. To rectify this situation, traditional chemical-by-chemical risk assessments must expand to incorporate consideration of combined health effects from exposure to a diverse array of environmental agents such as people encounter during their normal daily routines.

Efforts are currently underway to develop and apply innovative methods that assess cumulative health risk, where “cumulative risk” refers to the combined threats from exposures via all relevant routes to multiple environmental stressors, including biological, chemical, physical, and psychosocial entities [3,4]. “Cumulative risk assessment” is defined as a science-policy tool for organizing and analyzing relevant scientific information to examine, characterize, and possible quantify the combined adverse effects on human health from exposure to a combination of environmental stressors [3,4]. While these definitions are generally agreed on in principle, in practice the term “cumulative risk assessment” is often used in one of two different ways; to mean either (a) evaluation of combined effects from a mixture of chemicals, usually with similar modes of toxic action and/or toxic endpoints [8,9,10,17,18,19,20,21,22,23,24] or (b) appraisal of combined effects from a mixture of chemical and nonchemical (usually psychosocial) stressors [2,3,4,15,16,25,26,27,28,29]. It is worthwhile to examine the evolution of cumulative risk assessment within the context of these two domains. 

## 2. Historical Perspective

Scientists and risk assessors are well aware that toxicity can be modified by exposure to multiple environmental agents [7]. There is substantial evidence, for example, that simultaneous exposure to tobacco smoke and asbestos [30] or radon [31] increases the risk of lung cancer multiplicatively compared to the additive effects of the individual agents combined. Likewise, the risk of hepatocellular carcinoma is increased by the interactive effects of hepatitis B infection and exposure to aflatoxin-contaminated food [32], toxicity to aquatic organism is increased by interactions between polycyclic aromatic hydrocarbons and ultraviolet light [33], risks of hearing loss are potentiated by exposure to both noise and toluene [34], and children of parents experiencing stress are more susceptible to viral infections [35]. However, most of the available scientific evidence involves relatively simple interactions between comparatively few constituents, so that application of cumulative risk assessment to real-world mixtures is hindered or precluded by a scarcity of appropriate data, paucity of mechanistic understanding, and shortage of verified analytical frameworks [4,7].

### 2.1. Cumulative Risk Assessment for Chemical Mixtures

Historically, most risk assessments described as “cumulative” have focused primarily on reasonably simple chemical mixtures [5,6,7,8,9,10,19,20,21,22,23,24], wherein the constituents share a common mode of toxic action, such as organophosphate pesticides [18,35,36,37,38] or phthalates [39], and/or have the same health endpoint, like cancer [40,41,42,43]. A driving force behind the development of methods for assessment of cumulative risk from chemical mixtures has been U.S. environmental regulations aimed at controlling releases of hazardous anthropogenic chemicals, along with associated efforts by EPA to conduct appropriate risk assessments in support of science-based regulatory decision making [1,2,3,4,7,24]. Some of the important U.S. milestones marking the evolution of cumulative risk assessment applied to mixtures of chemicals are summarized in Table 1.

In 1986, EPA published risk assessment guidelines [8] for chemical mixtures, which were subsequently updated in 2000 [9] and expanded in 2006 [10]. According to the guidelines, the first priority when evaluating health effects of chemical mixtures is to use evidence for the mixture of concern, when it is available. The next highest priority is to use information about a similar mixture and, if that is not available, then assessors are advised to evaluate pairwise interactions between mixture constituents. Finally, when none of the preceding information is available, the default option for constituent interactions is to assume dose additivity (for chemicals with the same mechanism of action) or response additivity (for chemicals with the same health endpoint but different modes of action). 

The National Research Council (NRC) published a report in 1993 [44], which recommended that all relevant dietary and nondietary (e.g., ambient and indoor air, dust, soil, pets) exposures should be considered when evaluating the potential risks to infants and children from pesticides. In 1996, the Food Quality Protection Act (FQPA) [45] was signed into law, mandating that EPA consider children’s cumulative risks when regulating pesticides. The EPA subsequently conducted several cumulative risk assessments for pesticides with similar modes of toxic action, including organophosphates [18], chloroacetanilides [36], triazines [37], and n-methyl carbamates [38]. Amendments to the Safe Drinking Water Act [46] were passed in 1996 requiring EPA to develop new approaches to chemical mixtures in drinking water that took account of “… the prospects for synergistic or antagonistic interactions that may affect the shape of the dose-response relationship of the individual chemicals …”. Subsequent water-related research and cumulative risk assessments have focused primarily on disinfection byproducts for chemicals with similar mechanisms of action [47].

In 1999, the International Life Sciences Institute issued a report [17] proposing a tiered framework for cumulative risk assessment and, in 2000, EPA published its supplementary guidance for cumulative risk assessment of chemical mixtures [9]. In 2002, the EPA published its first cumulative risk assessment for pesticides [18] as mandated by the FQPA, and released the first of four National-scale Air Toxics Assessment (NATA) reports [48,49,50,51], which can be used to estimate cumulative cancer risks from exposure to hazardous air pollutants [40,41,42,43].

**Table 1:** Selected U.S. Milestones in the Evolution of Cumulative Risk Assessment for Health Effects from Exposure to Chemical Mixtures.

Year	Milestone	Reference(s)
1986	U.S. Environmental Protection Agency: *Guidelines for the Health Risk Assessment of Chemical Mixtures*	[8]
1993	National Research Council/National Academy of Sciences: *Pesticides in the Diets of Infants and Children*	[44]
1996	U.S. Federal Law (special protection for children from cumulative risk of pesticides in food): Food Quality Protection Act	[45]
1996	U.S. Federal Law (cumulative risk analysis for chemical mixtures in drinking water): Amendments to the Safe Drinking Water Act	[46]
1999	International Life Sciences Institute: *A Framework for Cumulative Risk Assessment*	[17]
2000	U.S. Environmental Protection Agency: *Supplementary Guidance for Conducting Health Risk Assessment of Chemical Mixtures*	[9]
2002	U.S. Environmental Protection Agency: *Organophosphate Pesticides: Revised Cumulative Risk Assessment*	[18]
2002	U.S. Environmental Protection Agency: National-scale Air Toxics Assessment (updated in 2006, 2009, 2011)	[48,49,50,51]
2004	Agency for Toxic Substances and Disease Registry: *Guidance Manual for the Assessment of Joint Toxic Action of Chemical Mixtures*	[19]
2006	U.S. Environmental Protection Agency: *Considerations for Developing Alternative Health Risk Assessment Approaches for Addressing Multiple Chemicals, Exposures and Effects*	[10]
2007	U.S. Environmental Protection Agency: *Concepts, Methods, and Data Sources for Cumulative Health Risk Assessment of Multiple Chemicals, Exposures and Effects: A Resource Document*	[52]
2008	National Research Council/National Academy of Sciences: *Phthalates and Cumulative Risk Assessment*	[39]
2009	Minnesota Pollution Control Agency: *Cumulative Air Emissions Risk Analysis at the MPCA – Background Document*	[53]
2010	*International Journal of Toxicology*: collection of articles on cumulative risk assessment for chemicals	[54,55,56,57,58]

In 2004, the Agency for Toxic Substances and Disease Registry (ATSDR) released its guidance manual [19] for risk assessment of chemical mixtures to assists scientists in determining whether exposure to chemical mixtures at hazardous waste sites might impact public health. The EPA published its expanded guidelines for cumulative risk assessment of chemical mixtures [10] in 2006, and its report on concepts, methods, and data sources for cumulative risk assessment of chemical mixtures [52] in 2007.

The 2008 NRC report, *Phthalates and Cumulative Risk Assessment* [39], concluded that the health risks associate with phthalate exposure should be evaluated using a cumulative risk assessment and provided guidance on suitable approaches and methods. The Minnesota Pollution Control Agency (MPCA) released a background document on cumulative air emissions risk analysis [53] in 2009 that reviewed the regulatory requirement to consider cumulative effects and discussed the methodology used to conduct a cumulative assessment. In 2010, a collection of articles on cumulative risks/impacts from chemical mixtures by scientists at the California Environmental Protection Agency (CalEPA) was published in the *International Journal of Toxicology* [54,55,56,57,58]. Today, cumulative risk assessment for health effects from exposure to chemical mixtures continues to be an important policy issue, research question, and risk assessment challenge [2,7,22,23,24,39].

### 2.2. Cumulative Risk Assessment for Combinations of Chemical and Nonchemical Stressors

There is mounting concern that exclusive focus on chemicals is overly narrow, needlessly restrictive, and clearly inadequate to address the totality of cumulative health risks from people’s real-world exposures to a diverse and dynamic combination of both chemical and nonchemical stressors [2,3,4,13,14,15,16,25,26,27,28,29]. Consequently, efforts are underway to develop approaches and methods that incorporate nonchemical stressors, especially psychosocial factors (e.g., low income, meager education, substandard diet, unsafe neighborhoods, dilapidated housing, lack of access to health care), into cumulative risk assessments. As shown in Table 2, EPA officially recognized the need for a more holistic approach to cumulative risk assessment in 1997 [59] when its Science Policy Council said that “The practice of risk assessment within the Environmental Protection Agency (EPA) is evolving away from a focus on the potential of a single pollutant in one environmental medium for causing cancer toward integrated assessments involving suites of pollutants in several media that may cause a variety of adverse effects on humans …”. That same year marked the release of a report [60] by the President’s Council on Environmental Quality, *Considering Cumulative Effects under the National Environmental Policy Act*, which summarized principles and reviewed methods for incorporating analysis of cumulative effects into an environmental assessment (EA) or an environmental impact statement (EIS).

In 2003, EPA published its *Framework for Cumulative Risk Assessment* [3] to provide a basis for development of future guidelines promoting consistency in cumulative risk assessment across EPA offices and programs. The EPA framework provides a conceptual structure to identify the fundamental elements and basic principles of an organized process for conducting and evaluating assessments of cumulative risk, including discussion of theoretical issues, technical matters, key definitions, and implementation issues. It envisions cumulative risk assessments occurring in three interrelated and generally sequential phases: (a) planning, scoping, and problem formulation; (b) analysis and integration of hazard, exposure, and dose-response information; and (c) interpretation and risk characterization [3,4]. As a follow-up to EPA’s framework, in 2004 the National Environmental Justice Advisory Council (NEJAC) published a report [15], *Ensuring Risk Reduction in Communities with Multiple Stressors: Environmental Justice and Cumulative Risks/Impacts* that commended the agency for opening up the scope of risk assessments “… to include environmental, health, social, and cultural factors that are key to understanding community risk”. It recommended that EPA combine the new cumulative risk framework with a collaborative problem-solving approach to, among other things, (a) incorporate the concept of vulnerability, particularly its social and cultural aspects, into EPA’s strategies and plans, (b) integrate social, economic, cultural, and community health factors, especially those involving vulnerability, into EPA’s decision making, and (c) develop and implement methods for screening, targeting, and prioritizing communities at elevated cumulative risk [15].

**Table 2:** Selected U.S. Milestones in the Evolution of Cumulative Risk Assessment for Health Effects from Exposure to a Combination of Chemical and Nonchemical Stressors.

Year	Milestone	Reference(s)
1997	U.S. Environmental Protection Agency (Science Policy Council): *Guidance on Cumulative Risk Assessment*	[54]
1997	Council on Environmental Quality (Executive Office of the President): *Considering Cumulative Effects under the NEPA*	[55]
2003	U. S. Environmental Protection Agency: *Framework for Cumulative Risk Assessment*	[3]
2004	National Environmental Justice Advisory Council (NEJAC): *Ensuring Risk Reduction in Communities with Multiple Stressors*	[15]
2007	*Environmental Health Perspectives*: collection of articles on cumulative risk assessment approaches	[4,7,61,62,63]
2009	National Research Council/National Academy of Sciences: *Science and Decisions: Advancing Risk Assessment*	[2]
2009	New Jersey Department of Environmental Protection: *A Preliminary Screening Method to Estimate Cumulative Environmental Impacts*	[26]
2010	California Environmental Protection Agency: *Cumulative Impacts: Building a Scientific Foundation*	[27]
2010	National Environmental Justice Advisory Council (NEJAC): *Nationally Consistent Environmental Justice Screening Approaches*	[28]
2010	*Journal of Exposure Science and Environmental Epidemiology*: collection of articles on cumulative risk assessment methods	[64,65,66]
2011	*International Journal of Environmental Research & Public Health*: collection of articles on cumulative health risk assessment	[22,23,29,67,68,69,70,71]
2011	International Life Sciences Institute, Risk Assessment in the 21st Century (RISK21) Project, Cumulative Risk Project Area	[72]

In 2007, several articles [4,7,61,62,63] commissioned by EPA on crucial issues related to cumulative risk assessment (e.g., phased approaches, disparities in vulnerability, differential exposure and health effects, role of biomarkers) were published as a mini-monograph in *Environmental Health Perspectives*. The NRC’s 2009 report [2], *Science and Decisions: Advancing Risk Assessment*, expressed concern that conventional risk assessment’s narrow chemical-by-chemical focus does not accurately capture the actual risks experienced by people because it typically excludes consideration of exposure to multiple chemical and nonchemical stressors and other factors that could influence vulnerability. The NRC opined that omission of these factors may mean that information necessary to identify at-risk populations and discriminate among competing risk management options is missing from assessments. The NRC found that “Without additional modifications, risk assessment might become irrelevant in many decision contexts, and its application might exacerbate the credibility and communication gaps between risk assessors and stakeholders” [2]. Also in 2009, the New Jersey Department of Environmental Protection (NJDEP) released its report [26], *A Preliminary Screening Method to Estimate Cumulative Environmental Impacts*, which discusses the data and methods being used by NJDEP to develop a screening tool to identify communities at increased risk from cumulative environmental impacts.

In 2010, CalEPA published a report [27], *Cumulative Impacts: Building a Scientific Foundation*, summarizing its efforts to develop a screening methodology to evaluate cumulative impacts of multiple sources of pollution in specific communities or geographic areas. The 2010 NEJAC report [28], *Nationally Consistent Environmental Justice Screening Approaches*, discussed principles that should guide the use of screening tools for cumulative risks and/or impacts, and described instances where a nationally consistent screening method, like the EPA’s Environmental Justice Strategic Enforcement Tool, would be either appropriate or inappropriate. That same year, the *Journal of Exposure Science and Environmental Epidemiology* published a collection of articles [64,65,66] by EPA scientists that provided a survey of EPA methods, procedures, and tools for cumulative risk assessment of chemical and nonchemical stressors.

In 2011/2012, the *International Journal of Environmental Research and Public Health* published a special issue on cumulative health risk assessment [22,23,29,67,68,69,70,71], of which this article is a part, which discussed current approaches to evaluation of combined effects from multiple environmental stressors. Also in 2011, the International Life Sciences Institute kicked off a project called Risk Assessment in the 21st Century (RISK21) aimed at “creating a science-based approach for improving human health risk assessments” and “developing a viable mechanism for transitioning to novel approaches” [72]. A component of that effort is devoted to reviewing the critical scientific issues in cumulative risk assessment and evaluating strengths and weakness of various approaches. Overall, the evidence indicates that assessment of cumulative health risks from exposure to a combination of chemical and nonchemical stressors is becoming an important decision-making tool in the U.S. [2,4,16,26,27].

### 2.3. Cumulative Risk Assessment Activities Outside the United States

As summarized in Table 3, development and application of cumulative risk assessment, both for chemical mixtures and combinations of chemical and nonchemical agents, have also occurred under the auspices of Canada, the United Kingdom, Denmark, the European Union, and the World Health Organization (WHO). In 1999, the Canadian Environmental Assessment Agency published a guide for cumulative effects assessment [73] to aid practitioners responsible for preparing cumulative evaluations for submission to appropriate regulatory bodies. The 2002 report [74], *Risk Assessment of Mixtures of Pesticides and Similar Mixtures*, published by the United Kingdom (UK), Food Standards Agency, recommended, among other things, that “a scientific and systematic framework should be established to decide when it is appropriate to carry out combined risk assessments of exposures to more than one pesticide and/or veterinary medicine.” 

**Table 3:** Selected Milestones Outside the U.S. in the Evolution of Cumulative Risk Assessment for Combined Health Effects from Multiple Environmental Stressors.

Year	Milestone	Reference(s)
1999	Canadian Environmental Assessment Agency: *Cumulative Effects Assessment Practitioners’ Guide*	[73]
2002	United Kingdom, Food Standards Agency: *Risk Assessment of Mixtures of Pesticides and Similar Substances*	[74]
2002	Danish Veterinary and Food Administration: *Combined Actions of Pesticides in Food*	[75]
2003	Danish Veterinary and Food Administration: *Combined Actions and Interactions of Chemicals in Mixtures*	[76]
2004	European Union, Integrated Research Project: Novel Methods for Integrated Risk Assessment of Cumulative Stressors in Europe (NoMiracle) – Project Period: 2004 to 2009	[77,78]
2005	European Union, Regulation No. 396/2005: Maximum Residue Levels of Pesticides in or on Food and Feed of Plant and Animal Origin	[79]
2007	European Union, European Food Safety Authority: *Cumulative Risk Assessment of Pesticides to Human Health*	[80]
2007	United Kingdom, Environment Agency: *Addressing Environmental Inequities: Cumulative Environmental Impacts*	[25]
2007	Canadian Environmental Assessment Agency (policy statement): *Addressing Cumulative Environmental Effects under the Canadian Environmental Assessment Act*	[81]
2008	World Health Organization: *Urban Heart: Health Equity Assessment & Response Tool* (update 2010 and user manual 2010)	[82,83,84]
2009	United Kingdom, Institute of Environment and Health: *Chemical Mixtures: A Framework for Assessing Risk to Human Health*	[85]
2009	World Health Organization/International Program on Chemical Safety: *Assessment of Combined Exposures to Multiple Chemicals*	[20,21]

The 2002 Danish report [75], *Combined Actions of Pesticides in Food*, examined whether there is a scientific basis for using a general standard formula in the risk assessment of pesticide mixtures, while the 2003 report [76], *Combined Actions and Interactions of Chemicals in Mixtures*, summarized concepts and discussed various approaches for risk assessment of chemical mixtures.

In 2004, the European Union (EU) initiated a 5-year, integrated research project [77,78] called Novel Methods for Integrated Risk Assessment of Cumulative Stressors in Europe (NoMiracle). The project involved 38 institutions from 17 countries, and the aim was to address the “… urgent need for development of methods for assessing the cumulative risks from combined exposures to multiple stressors including from complex mixtures of chemical, physical, and biological agents.” The main goal was to “… deliver understanding and tools for sound risk assessment, developing a research framework for the description and interpretation of combined stressor effects that leads to the identification of biomarkers and other indicators of cumulative impacts.” In 2005, the EU’s regulation number 396/2005, Maximum Residue Levels of Pesticides in or on Food and Feed of Plant and Animal Origin [79], stated that “In view of human exposure to combinations of active substances and their cumulative and possible aggregate and synergistic effects on human health ..” it is important “… to develop a methodology to take into account cumulative and synergistic effects.” 

The European Food Safety Agency published a report [80] in 2007, *Cumulative Risk Assessment of Pesticides to Human Health: The Way Forward*, which summarized results of a scientific colloquium to evaluate existing methodologies and identify new approaches. That same year, the UK Environment Agency released a report [25], *Addressing Environmental Inequalities: Cumulative Environmental Impacts*, noting that “There is a supportive political and policy context for taking forward work addressing cumulative environmental impacts.” Among the report’s recommendations were involvement of affected communities in understanding and assessing cumulative impacts, initiation of research on cumulative environmental impacts and inequalities, and evaluation of cumulative risk assessment tools. Also in 2007, the Canadian Environmental Assessment Agency released an updated policy statement [81], *Addressing Cumulative Environmental Effects under the Canadian Environmental Assessment Act*, which provided guidance on how responsible authorities should consider cumulative environmental effects in environmental assessments conducted under the Canadian Environmental Assessment Act.

In 2008, the WHO published *Urban HEART: Urban Health Equity Assessment and Response Tool*, which described a method for identifying and analyzing health disparities in urban environments [82]. An Urban HEART update [83] and a user manual [84] were published in 2010. In 2009, the UK’s Institute of Environment and Health published a report by the Interdepartmental Group on Health Risks from Chemicals, titled *Chemical Mixtures: A Framework for Assessing Risks to Human Health* [84], which concluded that “… chemical mixtures are best considered as a series of discrete, precisely defined problems for which clear boundaries can be set.” Also in 2009, the WHO, in collaboration with the International Program on Chemical Safety (IPCS), published *Assessment of Combined Exposures to Multiple Chemicals* [20], which summarized results of an international workshop and proposed a tiered framework to evaluate health effects from exposure to multiple chemicals. The WHO/IPCS framework, along with case studies on polybrominated diphenyl ethers and carbamates, was later published in the peer-reviewed literature [21].

## 3. Stressor-Based and Effects-Based Approaches to Cumulative Risk Assessment

Over the past twenty-five years, various forms of cumulative risk assessment have been applied with ever-increasing frequency to a diversity of situations and circumstances in the U.S. and elsewhere. Two general approaches [2,61] have emerged to evaluate combined health effects from exposure to multiple environmental factors: stressor-based methods used primarily for chemical mixtures and effects-based techniques typically applied to combinations of chemical and nonchemical stressors. The major differences between these complementary approaches are summarized in Table 4 [2,61].

**Table 4:** Important Differences between Stressor-Based (Bottom-up) and Effects-Based (Top-Down) Approaches to Cumulative Risk Assessment.

Attribute	Stressor-Based Approach	Effects-Based Approach
Analytical Strategy	Prospective, bottom-up analysis (evaluate constituent interactions)	Retrospective, top-down analysis (deconstruct and elucidate outcomes)
Central Question	What health effects are associated with a defined set of stressors?	Which stressors explain observed or hypothesized health outcomes?
Starting Point	Identification of key stressors and recognition of the populations and health end points influenced by them	Development of a conceptual model incorporating the stressors plausibly associated with critical health outcomes
Primary Emphasis	Analysis of stressor interactions to predict likelihood and severity of future adverse health outcomes	Determination of stressor contributions to observed or hypothesized health outcomes, including consideration of co-exposures and background processes
Typical Applications	Chemical mixtures	Combinations of chemical and nonchemical stressors
Driving Force(s)	Regulatory decisions about protection of human health from exposure to multiple chemicals [8,9,10,18,36,37,38,39,44,45,46]	Demands for “environmental justice”, concerns about health disparities, and calls for community-based risk assessments [3,4,11,12,13,14,15,16,28,64,65,66,77,78,79]

In essence, stressor-based (bottom-up) methods attempt to answer prospectively the question of “what health effects are related to a defined set of stressors?” by examining interactive effects among mixture constituents. Their development has been driven by regulatory questions about exposure to multiple chemicals and they are usually applied to chemical mixtures. Effects-based (top-down) approaches, on the other hand, aim to answer retrospectively the question of “which stressors explain observed or hypothesized health effects in a population or community?” by evaluating the cumulative effect of both chemical and nonchemical (usually psychosocial) stressors. Advances in effects-based techniques have been motivated by the need to evaluate “environmental” justice issues, explain reasons for observed health disparities, and respond to calls for community-based risk assessments.

Several different approaches for both stressor-based and effects-based cumulative risk assessment have been proposed, but there is no general consensus regarding appropriate methodologies or how best to structure appraisals to accomplish stated goals and objectives. Choice of an apt approach and decisions about related processes and procedures are dependent on the nature of the problem and the decision context within which it is being addressed [2,4]. Menzie *et al*. [61] have proposed a phased approach (also referred to as a tiered or iterative evaluation) for both stressor- and effects-based cumulative risk assessments. A comparison of various steps in each type of phased evaluation is presented in Table 5.

**Table 5:** Comparison of Phased Approaches for Stressor-Based and Effects-Based Cumulative Risk Assessment as described by Menzie *et al*. [61].

Phase	Stressor-Based Approach	Effects-Based Approach
Step 1	- develop conceptual model describing stressors and ways they cause effects - identify receptors and end points affected by stressors individually and in combination - establish common denominators for evaluation by identifying common receptors and end points	- develop conceptual model describing important stressors and the ways they cause critical effects - establish common denominators for evaluation by identifying common receptors and end points
Step 2	- screen stressors of interest to determine which need to be included in the assessment and which may act in combination	- screen potential stressors to identify an appropriate and manageable number to characterize the problem adequately
Step 3	- appraise individual effects of stressors along with combinations of other stressors as part of the conceptual model - determine how the combined effects of multiple stressors affect end point - incorporate psychosocial stressors by characterizing the environmental and cultural and socioeconomic attributes of exposed groups	- appraise the individual effects of individual stressors to determine whether one or a few stressors are predominant
Step 4	- assess the combined effect of stressors, taking into account potential interactions among the stressors and effects	- assess the combined effects of stressors without considering the potential for interactions
Step 5	- not applicable	- gauge the combined effect of stressors, taking into account potential interactions among the stressors and effects

Although overarching analytical frameworks, like those summarized in Table 5, offer general guidance on ways to systematically evaluate cumulative risk, we often lack adequate scientific knowledge and understanding about exposures, health effects, and the link between exposure and effects to implement them fully. The reality is that quantitative analyses are impractical in the context of many real-world problems because data on interactions among environmental stressors are scarce, information on place- and population-specific exposures is lacking, and verified mechanistic models relating exposure to effect are unavailable [2,4,7]. Thus risk assessors and risk managers are currently faced with reconciling two conflicting realities – escalating recognition of the need to conduct cumulative risk assessments as part of an informed decision-making process and growing appreciation of the inherent analytical complexities and substantial data deficiencies that hinder evaluation of cumulative health risks. 

To reduce some of the analytical challenges, the NRC [2] recently recommended modifying conventional approaches for cumulative risk assessment by orienting them around evaluation of risk management options instead of characterization of problems. The NRC felt that cumulative risk assessments would be most valuable to decision makers and affected communities when they “… can provide information about the health implications of alternative control options.” They cited the example of a community choosing among alternative methods for drinking-water disinfection; noting that it would be important to consider (a) potential health effects of changes in all disinfection by-products jointly, (b) simultaneous exposure to diverse waterborne pathogens, (c) all routes of exposure to key compounds of interest, and (d) vulnerable populations. The NRC pointed out that although many of the analytic tools would be similar, the focus on risk management options would change the decision context so that different factors might be correlated or affected on the margin compared to the typical consideration of baseline conditions, and different stressors might be deemed important. For example, stressors would only be included in the cumulative risk assessment to the degree that they influence the estimated benefits of a control option either in its estimation or interpretation [2]. The NRC’s modified version of the Menzie *et al*. [61] stressor-based approach is summarized in Table 6.

According to the NRC [2], this modified approach has several benefits, including: (a) explicit acknowledgement of stakeholder involvement in every phase of the assessment; (b) evaluation of background exposures and vulnerability factors can provide information relevant to environmental justice evaluations (focused on outcome inequality), which promotes the use of a single analytical framework for both risk assessment and environmental justice; (c) geospatial results from exposure and vulnerability assessments can be mapped to provide stakeholders with crucial and easy-to-understand information; and (d) detailed analysis and structured modeling are only necessary for a subset of stressors, which are circumscribed by their relevance to evaluation of costs and benefits for risk management options – other stressors might contribute to better understanding of background processes but otherwise would not need to be characterized quantitatively. Yet regardless of the benefits, daunting challenges persist. It is clear, for instance, that the scope and complexity of cumulative exposure to chemical and nonchemical stressors can easily surpass the capability of both stressor-based and effects-based evaluations to provide reliable quantitative estimates of cumulative health risk.

**Table 6:** The National Research Council’s Modified Version of the Stressor-Based Approach to Cumulative Risk Assessment, where the Primary Goal is to Discriminate among Risk Management Options [2].

Phase	Modified Stressor-Based Approach (Focus on Evaluation and Comparison of Risk Management Options)
Step 1	- develop conceptual model describing stressors and ways they cause effects, emphasizing those that would be significantly influenced by risk management option under study - identify receptors and end points affected by these stressors - review the conceptual model, including stressors, receptors, and end points with stakeholders as part of initial planning and scoping
Step 2	- use available scientific evidence and screening-level benefit calculations to make initial determination of which stressors should be included - review and re-evaluate planning and scoping activities based on stakeholder feedback - focus only on stressors that contribute to end points of interest for risk management options and are either differentially affected by various risk management options or influence the benefits of stressors that are differentially affected
Step 3	- evaluate the benefits of different risk management options with appropriate characterization of uncertainty, including quantification of the effects of individual stressors and bounding calculations of any possible interaction effects
Step 4	- conclude the analysis if results of Step 3 are sufficient to discriminate among risk management options given other economic, social, and political factors; otherwise, sequentially refine the analysis as needed, taking into account potential interactions among stressors

The NRC has noted that because of the number and variety of potentially important stressors, along with the diversity of community settings and population characteristics associated with differential vulnerability, there is a distinct possibility that cumulative risk assessments can become “… analytically intractable and therefore uninformative for making decisions in a timely fashion” [2]. Consequently, there is a need for simple methods to determine whether more refined methods are appropriate and whether information is adequate to inform risk management decisions. “The critical issue is to ensure that any simplified methods used in the context of cumulative risk assessment retain the key attributes of quantitative risk assessment, that is, consideration of both exposure and toxicity, notions of probability rather than just possibility, and information about the severity of health effects” [2]. While simplified methods may help to make cumulative risk assessment more practical; ultimately, decisions about whether to use stressor-based or effects-based approaches for assessment of cumulative health risks will depend on the questions being asked, the way in which the problem is framed, the decision context, and the geographical scale of analysis.

## 4. Summary and Conclusions

According to numerous national and international organizations, including the NRC [2], EPA [3], the National Environmental Justice Advisory Council [15,28], the states of New Jersey [26] and California [27], the UK Environment Agency [25], and the WHO [82,83,84], application of cumulative risk assessment to real-world problems is meant to broaden the extent of scientific analysis to incorporate psychological and sociological sources of stress so as to make risk analysis more (a) realistic in the sense of embodying actual, real-life situations and circumstances, (b) reliable as input to risk management decisions, (c) relevant to the problems confronting elected officials and regulatory decision makers, and (d) responsive to stakeholder concerns [16]. Cumulative risk assessments are intended to answer difficult and formerly unaddressed questions regarding combined risk burdens and disproportionate health impacts. As a result, they tend to be more theoretically complex, methodologically complicated, and computationally challenging than traditional single-chemical assessments [2,3,4]. 

Assessment of cumulative risk differs from conventional assessments in several important ways [2]: it involves evaluation of collective health effects of multiple stressors – as opposed to individual effects of a single stressor; it broadens the spectrum of environmental agents being appraised to include psychological (e.g., residential crowding) and sociological (e.g., racial discrimination) stressors – not just chemicals; it incorporates the concept of vulnerability (*i.e.*, differential biological susceptibility and exposure, as well as differential preparedness to withstand stressor effects and ability to recover from stressor effects) into the assessment explicitly – rather than treating it implicitly as is done in most conventional assessments; it focuses on population-based or location-based assessments of real-world cumulative exposures experienced by actual people – most conventional assessments entail source-based assessments of hypothetical people and theoretical exposures; it recognizes that the details (e.g., co-exposure to multiple agents, timing of exposure) and history (e.g., continuous versus intermittent, simultaneous versus sequential) of exposure to multiple stressors may be important for predicting risk – conventional assessments typically assume adverse effects are related solely to a combination of duration and intensity; it takes account of background exposures (*i.e.*, combined exposure to toxicologically relevant environmental stressors that are not necessarily the focus of the assessment), which may contribute to the cumulative risk under consideration – not normally evaluated as part of conventional risk assessments; and it provides for the possibility, depending on the circumstances, of a semi-quantitative or qualitative analysis/result – in contrast to most previous assessments, which are quantitative [16].

In point of fact, relatively few cumulative risk assessments have been conducted, and requisite conceptual models, theoretical frameworks, and analytical procedures are still being developed. The incorporation of nonchemical stressors, like discrimination and poverty, has been especially problematic; so much so that, to date, EPA has not included psychosocial stressors as part of any formal risk assessments [2]. The situation is likely to change in the future, however, as new methods and tools [22,23,26,27,28,61,64,65,66,81,82,83,84,86,87,88,89] as well as more rigorous theoretical paradigms and analytical frameworks [2,3,4,90,91,92,93,94,95] become available. For the present, we cannot let analytical complexities and associated scientific uncertainties deter us from moving forward with applications of cumulative risk assessment, even if they are incomplete or flawed. Better to go ahead with partial data, nascent procedures and screening-level approaches so that we can learn from our mistakes; improving both the process and its products through trial and error. It is important to remember that conducting a cumulative risk assessment, however imperfectly, does more than produce a risk estimate. It also organizes relevant scientific information, makes explicit the critical underlying assumptions and associated scientific uncertainties, provides a vehicle for framing important risk-related questions, and structures the debate about how to address them. In the end, it is always preferable to answer the right question, if only imprecisely, rather than answer the wrong question definitively. 
